# Sex Differences in Cardiovascular Risk and Diabetic Polyneuropathy: A Single-Center Retrospective Study in North-Eastern Hungary

**DOI:** 10.3390/jcm14165780

**Published:** 2025-08-15

**Authors:** Ferenc Sztanek, Attila Pető, László Imre Tóth, Hajnalka Lőrincz, Ágnes Molnár, Miklós Lukács, Adrienn Menyhárt, Péter Kempler, György Paragh, Mariann Harangi, Attila Csaba Nagy

**Affiliations:** 1 Department of Internal Medicine, Faculty of Medicine, University of Debrecen, H-4032 Debrecen, Hungary; toth.laszlo@med.unideb.hu (L.I.T.); lorincz_hajnalka@belklinika.com (H.L.); molnar.antalne@med.unideb.hu (Á.M.); paragh@belklinika.com (G.P.); harangi@belklinika.com (M.H.); 2 Third Department of Internal Medicine, Semmelweis Hospital of Borsod-Abauj-Zemplen County Central Hospital and University Teaching Hospital, H-3515 Miskolc, Hungary; petoattilaa@gmail.com (A.P.); doktorlumi@hotmail.com (M.L.); 3 Doctoral School of Health Sciences, University of Debrecen, H-4032 Debrecen, Hungary; 4 Department of Internal Medicine and Oncology, Faculty of Medicine, Semmelweis University, H-1085 Budapest, Hungary; menyhartadri@gmail.com (A.M.); kempler.peter@semmelweis.hu (P.K.); 5 Institute of Health Studies, Faculty of Health Sciences, University of Debrecen, H-4032 Debrecen, Hungary; 6 ELKH-UD Vascular Pathophysiology Research Group 11003, University of Debrecen, H-4032 Debrecen, Hungary; 7 Department of Health Informatics, Faculty of Health Sciences, University of Debrecen, H-4028 Debrecen, Hungary; attilanagy@med.unideb.hu

**Keywords:** diabetic sensorimotor polyneuropathy, cardiovascular risk factors, sex differences, type 2 diabetes mellitus

## Abstract

**Background/Objectives**: Diabetic sensorimotor polyneuropathy (DSPN) is a frequent microvascular complication of diabetes mellitus, associated with increased morbidity and reduced quality of life. The existing literature offers a limited understanding of sex-specific cardiovascular risk profiles and their association with DSPN, particularly within Central and Eastern European populations. **Methods**: A retrospective analysis was conducted using data from 621 individuals with type 1 or type 2 diabetes mellitus who underwent comprehensive neuropathy screening at the University of Debrecen between 2017 and 2021. The diagnosis of DSPN was made in accordance with international criteria, incorporating symptom scores, and electrophysiological measurements. Multivariate logistic regression was applied in order to identify independent predictors. **Results**: The diagnosis of DSPN was made in 444 individuals (71.5%), of whom 58.2% were female. Despite similar glycemic control (HbA1c: 7.81% in men vs. 7.65% in women, *p* = 0.297), men had significantly more frequent occurrences of previous myocardial infarction (11.8% vs. 5.0%, *p* = 0.008), peripheral vascular disease (19.9% vs. 12.7%, *p* = 0.041) and atherosclerosis (31.7% vs. 22.0%, *p* = 0.021). Multivariate analysis showed that female gender was independently associated with a lower incidence of DSPN (odds ratio [OR] = 0.592, 95% confidence interval [CI]: 0.369–0.950, *p* = 0.030), while diabetic retinopathy was a significant predictor (OR = 2.728, 95% CI: 1.300–5.725, *p* = 0.008). Electrophysiological testing revealed lower nerve conduction amplitudes in females for selected nerves. **Conclusions**: Our findings highlight sex-specific differences in neuropathy risk and support the implementation of individualized screening strategies in diabetic populations with region-specific risk factors.

## 1. Introduction

Diabetic neuropathy is considered one of the earliest and most common microvascular complications of diabetes, with a significant impact on global public health systems and healthcare expenditure [1]. The global prevalence of neuropathy among people with diabetes is estimated to range from 20% to 50% [2,3]. The Diabetes Control and Complications Trial showed that approximately 20% of people with type 1 diabetes (T1D) develop diabetic sensorimotor polyneuropathy (DSPN) within 20 years of disease onset [4]. In patients with newly diagnosed type 2 diabetes (T2D), the prevalence of DSPN is estimated to be 10–15% [5], increasing to nearly 50% after a decade of disease progression [6]. International guidelines recommend that screening for symptoms and clinical signs of DSPN be initiated at the time of T2D diagnosis, followed by annual reassessment [7,8]. Clinically, DSPN is characterized by rest pain in the lower extremities, paresthesia, and sensory deficits. These factors have been identified as major contributors to the development of diabetic foot ulcers and the potential need for lower limb amputation. These outcomes are associated with a marked decline in quality of life and increased mortality risk [1]. The principal risk factors for DSPN include a longer duration of diabetes and poor glycemic control. Achieving optimal glycemic regulation is therefore a cornerstone of preventive strategy [7]. Additional risk determinants, including age, sex, diabetic retinopathy, and modifiable cardiovascular risk factors (e.g., smoking, hypertension, dyslipidemia), have also been implicated in the pathogenesis of DSPN [9,10].

A fundamental element of clinical practice is the significance of community-based interventions spearheaded by health professionals and patient-provider communication in enhancing glycemic control and DSPN outcomes. The findings of research in this field indicate that nurse-led and community-based support programs have the capacity to significantly reduce HbA1c levels in patients with DSPN. These programs have the potential to serve as a valuable non-pharmacological adjunct to standard DSPN care, underscoring the significance of ongoing patient follow-up in clinical practice [11]. It has been demonstrated by other research that the importance of health literacy and education, as well as communication with health providers, in shaping patients’ self-care habits, is also emphasised by research on diabetes and DSPN. Patients who exhibited higher levels of health literacy and superior communication with their healthcare providers were more likely to adopt effective self-care practices during DSPN care [12]. The incorporation of these community-based factors, guided by health professionals, has the potential to enhance both clinical care and research, particularly in populations where disparities in health communication and educational access persist.

Emerging evidence highlights the role of biological sex as a significant modifier of DSPN risk, symptomatology and progression, though this is often underrecognized. A body of research in the field of epidemiology has indicated that males suffering from diabetes mellitus have a propensity to develop DSPN at an earlier age than is typical, and to display elevated levels of asymptomatic (subclinical) neuropathy and concomitant microvascular complications (for example, nephropathy and retinopathy). In contrast, women are significantly affected by painful neuropathic symptoms, particularly in the context of both T1D and T2D [13,14]. In the EURODIAB study, female sex emerged as an independent risk factor for incident painful DSPN in T1D, with an adjusted odds ratio of approximately 2.7 [15]. Moreover, in long-standing diabetes, women report greater neuropathic pain severity, a pattern likely mediated by a combination of hormonal, immune, and psychosocial factors, including postmenopausal estrogen decline and heightened inflammatory responses [14,16].

Objective assessments, including vibration perception threshold testing, nerve conduction studies, and standardized symptom questionnaires, remain fundamental components of the clinical evaluation of DSPN [17,18]. However, reliance on standardized diagnostic thresholds may result in the oversight of clinically relevant differences in symptomatology between sexes. Although not a standard indication, skin biopsies for intraepidermal nerve fiber density and electrophysiological testing can provide valuable diagnostic insight in cases presenting with atypical features or when alternative etiologies are suspected [1,19].

To date, studies examining cardiovascular risk and DSPN in diabetic populations have largely neglected to stratify results by sex. This is of particular pertinence given that both DSPN and cardiovascular risk factors are independently associated with increased all-cause mortality in different genders [20]. Although prior Hungarian studies have confirmed an association between DSPN and cardiovascular mortality [20,21], the sex-specific interplay between cardiovascular risk burden and neuropathy remains poorly understood. Given the known sex differences in pain perception, metabolic profiles, and neuropathy subtypes, this gap highlights an urgent need for focused investigations in region-specific diabetic populations, such as those in North-Eastern Hungary.

Accordingly, the present study undertakes a retrospective analysis of neuropathy screening data collected at the Diabetic Neuropathy Center, Department of Internal Medicine, Faculty of Medicine, University of Debrecen (2017–2021). The primary objectives of the study were to investigate the relationship between cardiovascular risk factors and microvascular complications in individuals with diabetes, with a particular focus on DSPN. The secondary objectives were to examine the extent to which sex-specific factors may influence these associations, thereby contributing to a understanding of disease progression and risk stratification. Accordingly, the central research question was to what extent do cardiovascular risk factors correlate with the presence and severity of DSPN, and how do these relationships differ between male and female patients with diabetes.

## 2. Materials and Methods

### 2.1. Study Population and Participant Enrollment

This manuscript was prepared in accordance with the STROBE (Strengthening the Reporting of Observational Studies in Epidemiology) guidelines for observational studies [22]. The corresponding completed STROBE checklist is available as a supplementary file (Supplementary File S1).

**Figure 1:**
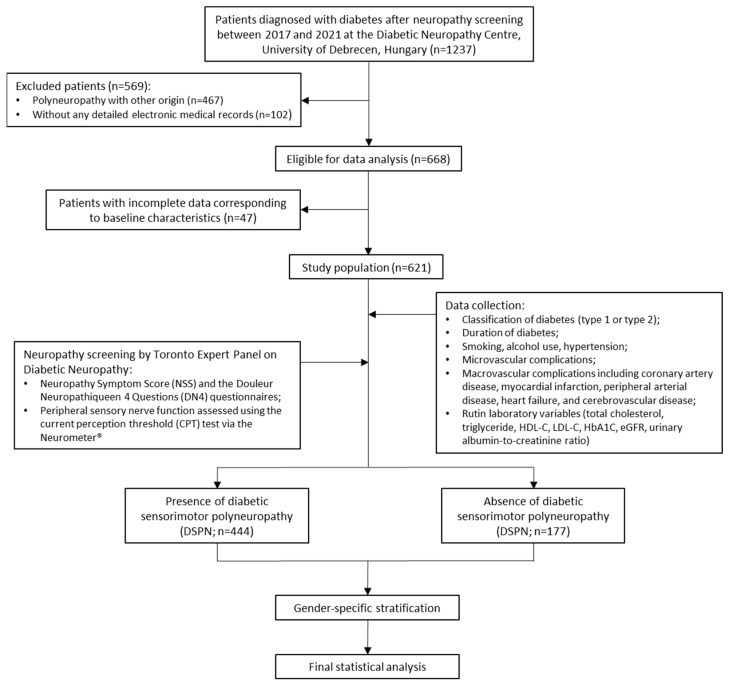
Study design flowchart of diabetic patients with and without diabetic sensorimotor polyneuropathy (DSPN).

The present retrospective study comprised a total of 1237 individuals with T2D or T1D who underwent standardized neuropathy screening at the Diabetic Neuropathy Centre, Department of Internal Medicine, Faculty of Medicine, University of Debrecen, between 1 January 2017 and 31 December 2021. In order to ensure diagnostic specificity, individuals with known alternative etiologies of polyneuropathy (n = 467) were excluded from the study. These etiologies included chronic alcohol consumption, hematological disorders, malignancy, chemotherapy exposure, autoimmune musculoskeletal conditions, or vitamin B12 deficiency. Patients lacking comprehensive electronic medical records (n = 102) or within complete baseline data (n = 47) were also excluded from the study. A total of 621 participants with confirmed DSPN and complete data sets were included in the final analysis (see Figure 1). All participants provided written informed consent. The study protocol adhered to the principles of the Declaration of Helsinki and received institutional ethical approval (protocol no. 5287-2/2019/EKU, approval date: 7 March 2019). Permission for research data access was granted between 1 April 2019, and 31 December 2022. The study design and reporting process were structured to meet internationally recognized transparency standards for retrospective cohort studies.

### 2.2. Data Collection and Variables Assessed

The extraction of demographic, clinical and laboratory data was facilitated by electronic health records and administrative databases from both hospital and general practice settings. The classification of diabetes mellitus was conducted in accordance with the International Classification of Diseases, Tenth Revision (ICD-10) codes E10 (T1D) and E11 (T2D), with a minimum disease duration of one year stipulated as an inclusion criterion.

The following baseline variables were collected: age, sex, type and duration of diabetes, smoking status, alcohol use, hypertension, and coexisting microvascular complications (diabetic retinopathy: E11.5; diabetic nephropathy: E11.2). Macrovascular comorbidities were also assessed, including coronary artery disease (I20, I24, I25), myocardial infarction (I21–I25.2), peripheral arterial disease (E11.5, I73.9), heart failure (I50), and cerebrovascular disease (I63, I64, G45). Hepatopathy included both nonalcoholic fatty liver disease (K76.0) and alcohol-induced liver injury (K70).

The laboratory variables encompassed hemoglobin A1c (HbA1c), estimated glomerular filtration rate (eGFR), urinary albumin-to-creatinine ratio, triglycerides, total cholesterol, low-density lipoprotein cholesterol (LDL-C), and high-density lipoprotein cholesterol (HDL-C). The pharmacological treatments administered were documented, encompassing oral antidiabetic agents, insulin utilization, and lipid-lowering therapies.

### 2.3. Sex-Specific Stratification

In view of the mounting evidence pointing to sex-specific patterns in DSPN, the data were stratified by biological sex (male vs. female) for all clinical, neurophysiological, and metabolic parameters. The objective of this stratification was to evaluate potential disparities in the prevalence and severity of painful versus painless DSPN phenotypes, metabolic risk profiles, and associated micro- and macrovascular complications. As has been demonstrated in previous studies, there is an earlier onset of painless DSPN in men and a higher prevalence of painful neuropathic symptoms in women, which may be mediated by hormonal and immune factors [13,15,16]. The present analysis also sought to explore such disparities within the Hungarian diabetic population.

### 2.4. Neuropathy Screening and Diagnosis

The diagnosis of DSPN was made in accordance with the criteria established by the Toronto Expert Panel on Diabetic Neuropathy [7]. All participants underwent structured symptom assessment using the Neuropathy Symptom Score (NSS) and the Douleur Neuropathique en 4 Questions (DN4) questionnaire. The diagnostic criteria for DSPN were defined as a DN4 score of ≥4 or an NSS score of ≥3. Furthermore, superficial sensory function was evaluated using the 10 g monofilament test, vibration sense with a 128 Hz tuning fork, and thermal perception thresholds [1,19].

Peripheral sensory nerve function was further assessed using the current perception threshold (CPT) test via the Neurometer^®^ (Neurotron Inc., Baltimore, MD, USA, 2002). Although the CPT provides valuable insight into Aβ-, Aδ-, and C-fiber function, it was not used in isolation for diagnostic confirmation [23].

The final diagnosis of DSPN was made on the basis of a combination of neuropathic symptoms/signs and at least one pathological finding on quantitative sensory testing, vibration perception, or CPT, in accordance with international recommendations [1,7,19].

### 2.5. Statistical Analysis

Categorical variables were presented as frequencies and percentages. Associations between categorical variables were evaluated using Pearson’s chi-squared test or Fisher’s exact test when expected cell counts were low. The normality of continuous variables was assessed using the Shapiro–Wilk test. Continuous variables were described as medians with interquartile ranges (IQR), and group comparisons were performed using the Mann–Whitney U test or Kruskal–Wallis H test, as appropriate.

In the analysis of patients with DSPN, the presence of diabetic neuropathy was defined as the primary outcome variable. To investigate factors associated with DSPN, multivariate logistic regression analysis was employed. The multivariable model used DSPN status as the dependent variable, and adjusted odds ratios (ORs) with 95% confidence intervals (CIs) were reported.

Independent variables included metrics of glycemic control and metabolic function (HbA1c, duration of diabetes), indicators of microvascular complications (eGFR, albumin-to-creatinine ratio, diabetic retinopathy), hypertension and smoking status. The composite cardiovascular endpoint comprised nonfatal myocardial infarction, chronic ischemic heart disease, nonfatal stroke, and peripheral arterial disease.

To explore sex-specific effects, interaction terms between sex and key clinical variables (e.g., diabetes duration, HbA1c, cardiovascular risk factors) were included in the multivariate logistic regression analysis. Additionally, stratified logistic regression analyses were conducted separately for male and female subgroups to identify sex-dependent associations with DSPN. This allowed for differential effect estimation and improved identification of potentially distinct risk profiles by sex. A *p*-value of <0.05 was considered statistically significant in all analyses. All statistical analyses were conducted using Intercooled Stata version 18 (StataCorp LLC, College Station, TX, USA).

## 3. Results

Baseline characteristics of the study population are summarized in Table 1. A total of 621 participants with diabetes were included in the study, with a median age of 67.0 years (IQR: 60.0–74.0 years) and a median diabetes duration of 14.0 years (IQR: 6.0–27.0 years). The majority of participants were female (59.6%), most individuals had T2D (91.1%). Comorbid hepatopathy was present in 10.8% of the population. Hypertension was highly prevalent, affecting 86.3% of participants. Current or former smoking was reported by 12.6% of individuals. Diabetic retinopathy was documented in 6.3% of the cohort. Regarding cardiovascular comorbidities, 22.2% had ischemic heart disease, and 6.6% reported a history of acute myocardial infarction. A history of stroke was noted in 8.1%, while 14.2% had previously experienced heart failure. Peripheral artery disease was reported by 8.5% of participants, and 23.7% had a documented history of atherosclerosis. Overall, 31.6% of individuals had a history of cardiovascular disease. In terms of pharmacological management, 57.0% of participants were receiving insulin therapy, and 91.5% were treated with oral antidiabetic agents. Statin therapy was used by 82.3% of the study population. Median eGFR was 80.0 mL/min/1.73 m^2^ (IQR: 72.0–90.0). Glycemic control, as assessed by HbA1c, had a median value of 7.3% (IQR: 6.7–8.0%). Lipid profile parameters were as follows: median triglyceride level was 1.6 mmol/L (IQR: 1.1–2.5), total cholesterol was 4.6 mmol/L (IQR: 3.8–5.6), HDL-C was 1.3 mmol/L (IQR: 1.0–1.5), and LDL-C was 2.7 mmol/L (IQR: 1.9–3.3).

Comparison of clinical and laboratory characteristics of diabetic patients with DSPN by sex is presented in Table 2. Among the 444 patients diagnosed with DSPN, 259 (58.2%) were female. The median age of female patients was slightly higher than that of male patients (69.0 [62.0–76.0] vs. 67.0 [61.0–73.0] years, *p* = 0.058). The distribution of diabetes type did not differ significantly between sexes, with the majority of patients having T2D in both groups (90.8% in males vs. 91.5% in females, *p* = 0.799). Renal function, as indicated by eGFR, was comparable between male and female patients. Similarly, HbA1c values were not significantly different. Lipid profiles showed some sex-specific differences. Although triglyceride levels were comparable (*p* = 0.832), female patients had significantly higher total cholesterol (4.0 [4.0–6.0] vs. 4.0 [4.0–5.0] mmol/L, *p* < 0.001), HDL-C (1.0 [1.0–2.0] vs. 1.0 [1.0–1.0] mmol/L, *p* < 0.001), and LDL-C levels (3.0 [2.0–4.0] vs. 2.0 [2.0–3.0] mmol/L, *p* = 0.021) compared to males. Hepatopathy was more prevalent among male patients (18.8% vs. 6.1%, *p* < 0.001), while the frequency of dyslipidemia did not differ significantly. The use of insulin therapy was comparable between the sexes, as was the prevalence of hypertension and smoking. Diabetic retinopathy showed no significant sex difference (9.7% in males vs. 7.3% in females, *p* = 0.378). However, males had a significantly higher prevalence of prior myocardial infarction (11.8% vs. 5.0%, *p* = 0.008), peripheral vascular disease (19.9% vs. 12.7%, *p* = 0.041), and atherosclerosis (31.7% vs. 22.0%, *p* = 0.021). Alcohol consumption was reported significantly more often by male patients (14.7% vs. 2.0%, *p* < 0.001). Neuropathy symptom scores (NTSS and DN4) did not differ significantly between sexes. Electrophysiological measurements revealed lower nerve conduction amplitudes in females for the right n. peroneus (*p* = 0.015) and left n. medianus (*p* = 0.036), while values for the left n. peroneus and right n. medianus were not significantly different. Regarding clinical assessments of sensory function, no significant differences were found in monofilament testing between sexes. However, female patients exhibited better vibratory perception as measured by tuning fork tests on both the left (*p* = 0.015) and right sides (*p* = 0.017).

Table 3 presents the results of the multivariate logistic regression analysis conducted to identify the predictors of DSPN within the study population. Among the variables examined, diabetic retinopathy emerged as a significant independent predictor of DSPN, with an odds ratio of 2.7 (95% CI: 1.300–5.725, *p* = 0.008). Sex also showed a significant association, with female patients exhibiting lower odds of DSPN compared to males (OR: 0.592; 95% CI: 0.369–0.950; *p* = 0.030). Other factors, including age, duration of diabetes, HbA1c, albumin/creatinine ratio, hypertension, and smoking status, were not found to be statistically significant predictors in the adjusted model.

## 4. Discussion

To the best of our knowledge, this is the first study to investigate the prevalence and risk profile of DSPN in the Northeastern region of Hungary, with particular attention to cardiovascular comorbidities and emerging gender differences. A single-center retrospective study may provide useful information on the prevalence and risk profile of diabetes and the earliest, but still least studied, microvascular complication, diabetic neuropathy. The Northeastern region of Hungary has been identified as a region of particular concern with regard to the prevalence of T2D and cardiovascular comorbidities, which has given rise to concerns regarding the potential for significant emerging sex disparities in the manifestation of the disease [20,21]. The relationship between DSPN and microvascular outcomes, including diabetic retinopathy and nephropathy, has not yet been comprehensively investigated in this population. Multivariate logistic regression analysis was employed to identify factors independently associated with DSPN, including diabetic retinopathy. Our results suggest that the current diagnostic framework for DSPN should incorporate sex adaptation, particularly in terms of cardiovascular disease, electrophysiological cut-offs, and sensory symptom profiles. Women in our study had lower CPT values despite similar neuropathic symptoms, suggesting that sex-adjusted electrophysiological cutoffs may improve diagnostic accuracy. Personalized screening approaches, including earlier or more frequent neuropathy screening in men at higher risk for cardiovascular disease and in peri- and postmenopausal women, may improve early detection of DSPN and facilitate personalized treatment [15].

Our findings are consistent with mechanistic models proposed in previous studies, in which hyperglycemia, cumulative oxidative damage, endothelial dysfunction and vascular aging are identified as key contributors to neurodegeneration [24,25,26]. Despite the fact that DSPN patients have had diabetes for a longer period of time, the duration of the disease itself was not independently predictive of DSPN in the multivariate model, presumably due to the advanced stage of the disease in the cohort as a whole. It is noteworthy that commonly associated clinical parameters, such as diabetes duration or HbA1c, did not independently predict DSPN in the multivariate model employed in this study. While older age exhibited a tendency to be associated with DSPN in preliminary analyses, it was not an independent predictor in the multivariate model. The present study’s findings are consistent with the conclusions of earlier research, which reported inconsistent associations between sex and the overall risk of DSPN [27,28,29]. Despite the existence of literature describing sex differences in painful symptoms and autonomic dysfunction [15], our data did not reveal significant differences in symptom scores between male and female patients. However, the observed lower adjusted odds of DSPN in women may indicate potential sex-related differences in the manifestation of the disease. It is important to note that women may experience cardiovascular risk factors differently; postmenopausal changes may increase their susceptibility to autonomic neuropathy and small-fiber dysfunction, which often manifest as painful symptoms [13,30]. Consequently, while previous studies have shown sex differences in the manifestation of DSPN, our results do not directly confirm this distinction.

It is evident from extant literature that these sex differences are the result of complex interactions between biological (hormonal, neuroimmune) and psychosocial factors. The hormone oestrogen is recognised for its neuroprotective and vasodilating properties, thus it may provide resistance to nerve injury during the reproductive life of women. Nevertheless, it has been determined that the absence of this protection following menopause is a contributing factor to the exacerbation of neuropathic pain in women. In contrast, testosterone has been shown to possess antinociceptive properties, which may contribute to the reduced prevalence of painful symptoms observed in men despite comparable structural nerve damage [14]. Intriguingly, female patients exhibited significantly superior vibratory perception in tuning fork assessments, which may be indicative of disparities in subclinical sensory thresholds between sexes.

Although HbA1c levels did not independently predict the presence of DSPN in the adjusted model, previous studies have considered HbA1c to be a factor that correlates with neurodegenerative processes [21,31,32]. As previously demonstrated in a study [33], intensive glycemic control has been shown to effectively reduce the progression of neuropathy in T1D. However, its efficacy appears to be more limited in T2D. These results provide further evidence for the hypothesis that chronic hyperglycaemia plays a significant role in the pathophysiology of DSPN.

The observed association between a history of dyslipidemia and DSPN is consistent with previous reports, although no significant correlation was found with current plasma lipid levels. The widespread use of lipid-lowering agents could confound the correlation between in vivo lipid values and neuropathy risk. The existing literature indicates that elevated LDL-C and triglyceride levels, as well as reduced HDL-C levels, are contributing factors in the development of neuropathy [34,35,36]. Notwithstanding the finding that current plasma lipid levels, in particular total cholesterol, HDL-C, and LDL-C, were elevated among female patients, no direct correlation was identified between these parameters and DSPN in the present analysis. This finding indicates that, while dyslipidaemia has been implicated in DSPN pathogenesis in previous studies, the role of circulating lipid levels as independent predictors may be diminished by other factors, such as lipid-lowering therapy or metabolic differences unrelated to sex [37].

The traditional cardiovascular risk factors of hypertension and smoking were not found to be independent predictors in the multivariate model. Insulin therapy, which is often indicative of advanced diabetes or severe β-cell failure, similarly showed no sex difference or significant association with the prevalence of DSPN in the current cohort. This may be related to the multifactorial nature of T2D, where several clinical factors, including glycemic variability, treatment history, and comorbidities, may influence the risk of neuropathic complications. Further prospective studies are needed to examine these associations. This may indicate the multifactorial nature of T2D, where several overlapping factors—including glycemic variability, treatment history, and comorbidities—interact to modulate the risk of neuropathic complications. Further prospective studies are needed to explore these associations.

In our DSPN cohort, retinopathy was found to be a significant independent predictor of DSPN (OR = 2.728; *p* = 0.008), indicating a strong microvascular association. The association between DSPN and diabetic retinopathy in the present sample lends support to the concept of shared pathogenic pathways among microvascular complications, including chronic oxidative stress, activation of intracellular signaling cascades (polyol, hexosamine, protein kinase C), and impaired neurovascular regulation [38]. Although albuminuria was not an independent predictor of DSPN in our model, its known role in microvascular complications suggests a potential pathophysiological link [39,40,41,42]. Although eGFR was not significantly different between sexes or identified as a predictor, previous studies support its association with diabetic microangiopathy.

In the present study, cardiovascular comorbidities, such as myocardial infarction and peripheral arterial disease, were more common in male patients, which is consistent with the results of previous studies that found an association between DSPN and cardiovascular disease, and that this association may be mediated by autonomic nervous system dysfunction in addition to atherosclerotic vascular disease [43,44]. Reanalysis of the ACCORD study revealed sex-related differences, finding that self-reported neuropathy was associated with a twofold increase in mortality in the intensive treatment group. This association may reflect the impact of neuropathy-related autonomic dysfunction, which can lead to cardiac arrhythmias and impaired perfusion and ultimately result in heart failure [45,46]. Despite the absence of a direct assessment of autonomic outcomes in the present study, extant literature suggests that postmenopausal women may exhibit heightened susceptibility to cardiac autonomic neuropathy, a condition that may arise from estrogen deficiency [30].

The clinical implications of our findings are manifold. Retinopathy emerged as an independent predictor of DSPN, underscoring the value of integrated screening in patients with microvascular complications. Although symptom scores did not differ significantly by sex, women were less likely to have DSPN based on adjusted analysis, suggesting that screening strategies may benefit from sex-specific considerations. For example, earlier implementation of objective diagnostic tools, such as vibration threshold testing, may be particularly useful in men, while women—especially during the menopausal transition—may require increased attention to painful symptoms and signs of autonomic dysfunction [37]. The sensory spinal cord is particularly vulnerable to damage in diabetes, with nearly half of patients with T2D for more than 10 years developing some degree of peripheral neuropathy [6]. Recent ex vivo results using advanced magnetic resonance imaging techniques, such as diffusion tensor imaging, have revealed detectable structural changes, warranting further discussion of their potential role in future screening strategies [47,48]. Despite these differences, current diagnostic frameworks remain largely sex-neutral, potentially overlooking the complex biological and hormonal factors influencing neuropathy risk. Further research is needed to clarify sex-specific patterns in symptom expression and nerve function, particularly in relation to painful and autonomic neuropathies [13,14,15,30,37].

The present findings carry significant implications for the multidisciplinary clinical practice management of DSPN. The necessity to prioritise the integration of community-based, nurse-led interventions into routine care is emphasised, as evidence has demonstrated their efficacy in significantly reducing HbA1c levels and enhancing metabolic control in individuals with DSPN [11]. Health literacy, patient education, and improved patient-provider communication are all equally central to effective DSPN care. Patients with higher levels of health literacy and better communication with healthcare providers are more likely to adopt effective self-care behaviours. The incorporation of structured education and communication strategies into DSPN care has therefore been hypothesised to improve adherence to therapy, empower patients, and improve both symptom management and overall quality of life [12]. In regions such as parts of Central and Eastern Europe, where there are significant inequalities in access to and communication with healthcare, the incorporation of these approaches into existing screening and treatment frameworks would align DSPN care with international recommendations calling for multidisciplinary, patient-centred strategies. Furthermore, interventions should be tailored to account for gender-specific differences in symptom manifestation, autonomic function, and cardiovascular risk profiles, with a view to further improving diagnostic accuracy and treatment effectiveness.

Although the strengths of our study include the large sample size and standardized neuropathy assessments, several key limitations must be acknowledged. The retrospective, single-center design of the study, and the lack of detailed lifestyle, psychosocial, and autonomic data are limitations that are relevant to examining the prevalence of DSPN by sex. The single tertiary care setting may introduce selection bias, potentially overrepresenting more complex or refractory cases. The cross-sectional nature of the study precludes the possibility of drawing causal inferences. Furthermore, lifestyle factors, comorbidities, and medication use were not examined in detail. Based on registry data, hepatopathy in this study likely refers to nonalcoholic fatty liver disease; however, the lack of a clearly defined diagnostic framework limits its distinction from alcoholic liver disease, despite chronic alcohol consumption being an exclusion criterion. Although we excluded patients with confirmed vitamin B12 deficiency based on registry data, the absence of direct laboratory measurements of vitamin B12 and haematocrit levels, as well as the high prevalence of cardiovascular disease potentially related to the use of antiplatelet agents and proton pump inhibitors, means that we cannot rule out subclinical or undiagnosed vitamin B12 deficiency in this cohort. Finally, the single-center design may introduce selection bias, and the lack of longitudinal follow-up precludes the assessment of DSPN progression over time. Future multicenter studies, including comprehensive neurophysiological, autonomic, and hormonal studies, are needed to clarify the complex relationships in DSPN by sex distribution.

## 5. Conclusions

This study is the first to characterize the prevalence and risk profile of DSPN by gender in the Northeastern region of Hungary, a region burdened by diabetes and cardiometabolic diseases. The findings of this study corroborated the multifactorial character of DSPN and underscored the potential for sex-based disparities in the prevalence and presentation of neuropathy, particularly with regard to cardiovascular comorbidity and sensory thresholds, particularly in male patients. Retinopathy was identified as an independent predictor of DSPN, indicating a correlation with diabetic microvascular complications and emphasizing the necessity for their joint investigation. The lower and milder probability of DSPN in women may be attributable to hormonal or neuroimmune disparities. The findings of this study may indicate a necessity for sex-specific diagnostic strategies and adapted screening protocols, with the aim of taking into account both biological and regional variability in the care of DSPN patients. It is evident that the validation of these associations and the establishment of the basis for personalised interventions will require further prospective multicentre studies.

## Figures and Tables

**Table 1:** The baseline characteristics of the study population.

Variable	Study Population N = 621
Age (years), median [IQR]	67.0 [60.0−74.0]
Duration of diabetes (years), median [IQR]	14.0 [6.0–27.0]
Gender	
Male, n (%)	251 (40.4)
Female, n (%)	370 (59.6)
Type of diabetes	
T1D, n (%)	55 (8.7)
T2D, n (%)	566 (91.1)
Hepatopathy	
Yes, n (%)	67 (10.8)
No, n (%)	554 (89.2)
Hypertension	
Yes, n (%)	536 (86.3)
No, n (%)	85 (13.7)
Smoking	
Yes, n (%)	78 (12.6)
No, n (%)	543 (87.4)
Retinopathy	
Yes, n (%),	39 (6.3)
No, n (%)	582 (93.7)
Ischemic heart disease	
Yes, n (%)	138 (22.2)
No, n (%)	483 (77.8)
History of acute myocardial infarction	
Yes, n (%)	41 (6.6)
No, n (%)	580 (93.4)
History of stroke	
Yes, n (%)	50 (8.1)
No, n (%)	571 (92.0)
History of heart failure	
Yes, n (%)	90 (14.2)
No, n (%)	531 (85.5)
History of peripheral artery disease	
Yes, n (%)	53 (8.5)
No, n (%)	568 (91.5)
History of atherosclerosis	
Yes, n (%)	147 (23.7)
No, n (%)	474 (76.3)
History of cardiovascular disease	
Yes, n (%)	196 (31.6)
No, n (%)	425 (68.4)
Insulin treatment	
Yes, n (%)	354 (57.0)
No, n (%)	267 (43)
Oral antidiabetic drugs	
Yes, n (%)	568 (91.5)
No, n (%)	53 (8.5)
Statin treatment	
Yes, n (%)	511 (82.3)
No, n (%)	110 (17.7)
eGFR (ml/min/1.73 m^2^), median [IQR]	80.0 [72.0, 90.0]
HbA1c (%), median [IQR]	7.3 [6.7, 8.0]
Triglyceride (mmol/L), median [IQR]	1.6 [1.1, 2.5]
Total cholesterol (mmol/L), median [IQR]	4.6 [3.8, 5.6]
HDL-C (mmol/L), median [IQR]	1.3 [1.0, 1.5]
LDL-C (mmol/L), median [IQR]	2.7 [1.9, 3.3]

Legend: Continuous variables are presented as median [interquartile ranges; IQR], and categorical variables as number (percentage; %). T1D: type 1 diabetes; T2D: type 2 diabetes; eGFR: estimated glomerular filtration rate; HbA1c: glycated hemoglobin; HDL-C: high-density lipoprotein cholesterol; LDL-C: low-density lipoprotein cholesterol.

**Table 2:** The comparison of data for diabetic patients with DSPN by sex.

Variable	Male Patients with DSPN	Female Patients with DSPN	*p*-Value
	n = 185 (41.8%)	n = 259 (58.2%)	
			
Age (years), median [IQR]	67.0 [61.0, 73.0]	69.0 [62.0, 76.0]	0.058
Duration of diabetes (years), median [IQR]	14.0 [9.0, 21.0]	13.0 [7.0, 22.0]	0.271
Type of diabetes (total)			
T1D: n (%)	17 (9.19)	22 (8.49)	0.799
T2D: n (%)	168 (90.81)	237 (91.51)	
eGFR (ml/min/1.73m^2^), median [IQR]	76.5 [67.0, 84.0]	78.0 [69.0, 86.0]	0.255
HbA1c (%)	7.81 (±1.16)	7.65 (± 1.06)	0.297
Albumin/creatinine ratio (mg/mmol), median [IQR]	3.0 [2.0, 5.0]	3.0 [2.0, 5.0]	0.724
Triglyceride (mmol/L), median [IQR]	2.0 [1.0, 3.0]	2.0 [1.0, 3.0]	0.832
Total cholesterol (mmol/L), median [IQR]	4.0 [4.0, 5.0]	4.0 [4.0, 6.0]	<0.001
HDL-C (mmol/L), median [IQR]	1.0 [1.0, 1.0]	1.0 [1.0, 2.0]	<0.001
LDL-C (mmol/L), median [IQR]	2.0 [2.0, 3.0]	3.0 [2.0, 4.0]	0.021
Hepatopathy, n (%)	35 (18.8%)	16 (6.1%)	<0.001
Dyslipidemia, n (%)	150 (80.7%)	205 (79.2%)	0.699
Insulin treatment, n (%)	84 (45.2%)	126 (48.7%)	0.467
Hypertension, n (%)	169 (90.9%)	226 (87.6%)	0.279
Smoking, n (%)	30 (16.2%)	28 (11.0%)	0.113
Retinopathy, n (%)	18 (9.7%)	19 (7.3%)	0.378
Ischemic heart disease, n (%)	53 (28.5%)	65 (25.1%)	0.423
History of acute myocardial infarction, n (%)	22 (11.8%)	13(5.0%)	0.008
History of stroke, n (%)	12 (6.5%)	24 (9.3%)	0.283
History of heart failure, n (%)	36 (19.4%)	43 (16.6%)	0.454
History of peripheral vascular disease, n (%)	37 (19.9%)	33 (12.7%)	0.041
History of atherosclerosis, n (%)	59 (31.7%)	57 (22.0%)	0.021
Alcohol consumption, n (%)	27 (14.7%)	5 (2.0%)	<0.001
NTTS score, mean (SD)	11.23 (±5.98)	11.76 (±6.38)	0.387
DN4 score, mean (SD)	3.08 (±1.54)	3.18 (±1.62)	0.456
N. peroneus left (mA), median [IQR]	538.0 [415.0, 769.0]	495.5 [422.0, 604.0]	0.055
N. peroneus right (mA), median [IQR]	579.0 [446.0, 826.0]	535.0 [427.0, 668.0]	0.015
N. medianus left (mA), median [IQR]	293.5 [248.0, 346.0]	279.0 [239.0, 333.0]	0.036
N. medianus right (mA), median [IQR]	315.5 [262.0, 376.0]	305.0 [260.0, 373.0]	0.578
Monofilament test, left, n (%)	31 (16.67)	39 (15.12)	0.658
Monofilament test, right, n (%)	33 (17.74)	40 (15.50)	0.530
Tuning fork, left, median [IQR]	4.0 [1.0, 5.0]	4.0 [2.0, 5.0]	0.015
Tuning fork, right, median [IQR]	3.5 [1.0, 5.0]	4.0 [2.0, 5.0]	0.017

Legend: Continuous variables are presented as median [interquartile ranges; IQR] or mean ± standard deviation (SD), as appropriate, and categorical variables as number (percentage; %). *p*-values refer to comparisons between male and female diabetic sensorimotor polyneuropathy (DSPN) patients. T1D: type 1 diabetes; T2D: type 2 diabetes; eGFR: estimated glomerular filtration rate; HbA1c: glycated hemoglobin; HDL-C: high-density lipoprotein cholesterol; LDL-C: low-density lipoprotein cholesterol; NTSS: Neuropathy Total Symptom Score; DN4: Douleur Neuropathique 4 questionnaire.

**Table 3:** The results of the multivariate logistic regression analysis to explore the predictors of diabetic sensorimotor polyneuropathy (DSPN) within our study group.

Factors	Odds Ratio (OR)	95% Confidence Interval	*p*-Value
Age (years)	1.014	0.989–1.040	0.272
Gender (female/male)	0.592	0.369–0.950	0.030
Duration of diabetes (years)	1.020	0.995–1.045	0.111
HbA1c (%)	1.143	0.915–1.428	0.238
Albumin/creatinine ratio (mg/mmol)	0.988	0.970–1.007	0.202
Hypertension	2.202	0.842–5.758	0.108
Smoking	1.654	0.868–3.151	0.126
Retinopathy	2.728	1.300–5.725	0.008

Legend: HbA1c: glycated hemoglobin.

## Data Availability

The clinical and laboratory data used to support the findings of this study are included within the article.

## Supplementary Materials

The following supporting information can be downloaded at: https://www.mdpi.com/article/10.3390/jcm14165780/s1; Supplementary File S1: Strengthening the Reporting of Observational Studies in Epidemiology (STROBE) guidelines for observational studies.

## Abbreviations

The following abbreviations are used in this manuscript:

CIs	confidence intervals
CPT	current perception threshold
DN4	Douleur Neuropathique en 4 Questions
DSPN	diabetic sensorimotor polyneuropathy
eGFR	estimated glomerular filtration rate
HbA1c	hemoglobin A1c
HDL-C	high-density lipoprotein cholesterol
IQR	interquartile ranges
LDL-C	low-density lipoprotein cholesterol
NSS	Neuropathy Symptom Score
T1D	type 1 diabetes
T2D	type 2 diabetes
