# Supplementary material for: Sex Differences in Cardiovascular Risk and Diabetic Polyneuropathy: A Single-Center Retrospective Study in North-Eastern Hungary
Source: J Clin Med. 2025 Aug 15;14(16):5780. doi: 10.3390/jcm14165780 (PMC12386531; doi:10.3390/jcm14165780)
Supplement: Supplementary file 1 [file jcm-14-05780-s001.zip › jcm-3819804-supplementary.pdf]

**STROBE Checklist – Strengthening the Reporting of Observational Studies in Epidemiology**

**STROBE Checklist for Observational Studies – Filled for " Sex Differences in Cardiovascular Risk and Diabetic Polyneuropathy: A Single-Center Retrospective Study in North-Eastern Hungary" Study**

This study is reported in accordance with the STROBE (Strengthening the Reporting of Observational Studies in Epidemiology) guidelines for observational studies [von Elm et al., 2007]. The completed STROBE checklist is provided as Supplementary File 1 and follows the recommendations of the EQUATOR Network (https://www.equator-network.org/reporting-guidelines/strobe/).

**Checklist**

**Title and Abstract**

1. **Indicate the study's design** – Retrospective observational study indicated in the abstract and methods.
   *Location in manuscript: Title, Abstract, Methods 2.1*
2. **Provide an informative and balanced summary** – Summary of objectives, design, participants, main results included.
   *Location in manuscript: Abstract*

**Introduction**

3. **Background/rationale** – Background on DSPN prevalence, impact, and risk factors provided.
   *Location in manuscript: Introduction, first three paragraphs*
4. **Objectives** – Primary and secondary objectives clearly stated, including research question.
   *Location in manuscript: End of Introduction / Objectives section*

**Methods**

5. **Study design** – Retrospective design explicitly stated; inclusion/exclusion criteria provided.
   *Location in manuscript: Methods 2.1*
6. **Setting** – University of Debrecen, Diabetic Neuropathy Centre; study period: 1 Jan 2017–31 Dec 2021.
   *Location in manuscript: Methods 2.1*
7. **Participants** – Inclusion: confirmed T1DM/T2DM, ≥1 year disease duration; exclusion: alternative polyneuropathy causes, incomplete records.
   *Location in manuscript: Methods 2.1*
8. **Variables** – Clearly defined: DSPN diagnosis, cardiovascular risk factors, comorbidities, lab markers.
   *Location in manuscript: Methods 2.2, 2.4*
9. **Data sources/measurement** – Electronic health records, administrative databases; standardized neuropathy tests.
   *Location in manuscript: Methods 2.2, 2.4*
10. **Bias** – Diagnostic specificity ensured by exclusion criteria; standardized testing to minimize misclassification.
    *Location in manuscript: Methods 2.1, 2.4*
11. **Study size** – Initial N=1237, exclusions detailed, final N=621.
    *Location in manuscript: Methods 2.1, Figure 1*
12. **Quantitative variables** – Continuous variables described as medians (IQR), categorical as frequencies (%).
    *Location in manuscript: Methods 2.5*
13. **Statistical methods** – Chi-square, Fisher's exact, Shapiro–Wilk, Mann–Whitney U, Kruskal–Wallis, logistic regression; sex-stratified analyses.
    *Location in manuscript: Methods 2.5*

**Results**

14. **Participants** – Flowchart of participant inclusion/exclusion; numbers and reasons provided.
    *Location in manuscript: Figure 1, Results first paragraph*

15. **Descriptive data** – Baseline demographics, clinical characteristics, comorbidities described.
*Location in manuscript: Results, Table 1*

16. **Outcome data** – Prevalence of DSPN, painful vs painless phenotypes, sex-specific differences.
*Location in manuscript: Results section*

17. **Main results** – Adjusted odds ratios for DSPN risk factors reported; sex-specific findings highlighted.
*Location in manuscript: Results, Tables 2–3*

18. **Other analyses** – Interaction terms for sex; stratified regression analyses.
*Location in manuscript: Results, last part*

**Discussion**

19. **Key results** – Summary of main findings in context of objectives.
*Location in manuscript: Discussion opening paragraph*

20. **Limitations** – Retrospective design, potential residual confounding, reliance on available records.
*Location in manuscript: Discussion limitations paragraph*

21. **Interpretation** – Interpretation in context of existing literature; clinical implications discussed.
*Location in manuscript: Discussion main body*

22. **Generalisability** – Findings generalisable to similar Central/Eastern European diabetic populations.
*Location in manuscript: Discussion final paragraph*

**Other Information**

23. **Funding** – Funding source and role of funders stated.
*Location in manuscript: Acknowledgements/Funding statement*

*Reference: von Elm E, Altman DG, Egger M, Pocock SJ, Gøtzsche PC, Vandenbroucke JP; STROBE Initiative. The Strengthening the Reporting of Observational Studies in Epidemiology*

*(STROBE) statement: guidelines for reporting observational studies. Lancet. 2007;370(9596):1453-1457. doi:10.1016/S0140-6736(07)61602-X.*
